# Cell Type-Selective Expression of Circular RNAs in Human Pancreatic Islets

**DOI:** 10.3390/ncrna4040038

**Published:** 2018-11-27

**Authors:** Simranjeet Kaur, Aashiq H. Mirza, Flemming Pociot

**Affiliations:** 1 Steno Diabetes Center Copenhagen, 2820 Gentofte, Denmark; simranjeet.kaur@regionh.dk; 2 Weill Cornell Medicine, Department of Pharmacology, New York, NY 10065, USA; aah2003@med.cornell.edu; 3 Copenhagen Diabetes Research Center (CPH-DIRECT), Department of Pediatrics E, Herlev Hospital, 2730 Herlev, Denmark; 4 Faculty of Health and Medical Sciences, University of Copenhagen, DK-1165 Copenhagen, Denmark

**Keywords:** circular RNAs, circRNA, human islets, RNA-seq, type 1 diabetes, β-cell

## Abstract

Understanding distinct cell-type specific gene expression in human pancreatic islets is important for developing islet regeneration strategies and therapies to improve β-cell function in type 1 diabetes (T1D). While numerous transcriptome-wide studies on human islet cell-types have focused on protein-coding genes, the non-coding repertoire, such as long non-coding RNA, including circular RNAs, remains mostly unexplored. Here, we explored transcriptional landscape of human α-, β-, and exocrine cells from published total RNA sequencing (RNA-seq) datasets to identify circular RNAs (circRNAs). Our analysis revealed that circRNAs are highly abundant in both α- and β-cells. We identified 10,830 high-confidence circRNAs expressed in human α-, β-, and exocrine cells. The most highly expressed candidates were *MAN1A2*, *RMST*, and *HIPK3* across the three cell-types. Alternate circular isoforms were observed for circRNAs in the three cell-types, indicative of potential distinct functions. Highly selective α- and β-cell circRNAs were identified, which is suggestive of their potential role in regulating β-cell function.

## 1. Introduction

The emergence of non-coding RNAs as essential players in the regulation of gene expression has advanced our understanding of many cellular processes and provided novel mechanistic insights into various pathophysiological conditions [1]. In the last decade, non-coding RNAs, including microRNAs and long non-coding RNAs (lncRNAs), have been implicated in various diseases, such as cancers, neurological disorders, and autoimmune diseases [2,3,4,5,6]. Circular RNAs (circRNAs) are a repertoire of a newly discovered class of endogenous non-coding RNAs that are characterized by covalently linked 3′ and 5′ ends through a backsplicing process to form a closed loop circular structure, hence named circular RNAs. circRNAs are generally formed by alternative splicing of pre-mRNA and do not possess a 5′ cap or a 3′ Poly A tail, a characteristic feature of mRNAs [7,8,9,10,11].

circRNAs are a highly abundant, conserved, stable, and highly regulated phenomenon across many species [12,13]. Their ubiquitous as well as cell-type specific expression has been independently confirmed by high throughput sequencing of the ribonuclease (RNase) R treated RNA combined with informatics algorithms to characterize circRNAs by detecting back-spliced junctions [13,14]. A number of studies have shown that circRNAs are expressed independent of their cognate linear mRNA [11,15,16]. The circRNA levels (as measured by circRNA-specific junction counts) do not correlate with the overall abundance of the corresponding mRNA [11,14,15,16]. circRNAs have been found enriched in neuronal tissues and exhibit dynamic expression patterns during various developmental stages [12,17,18,19,20,21].

Although, in the past few years, the number of circRNAs with known biological functions has rapidly grown, for a large majority of circRNAs, their functions remain unknown. Several studies have shown circRNAs can function as competing endogenous RNAs (ceRNAs) or microRNA (miRNA) sponges [22,23,24]. For example, CDR1as or ciRS-7 contains more than 70 conserved binding sites for miRNA-7 [22,23] and is involved in regulating insulin content and secretion in mouse pancreatic islets [24]. circ-SRY is another important circRNA exclusively found expressed in mice testis and contains 16 binding sites for miRNA-138 [23]. Other examples of circRNAs known to act as miRNA sponges include: Circ-ITCH [25], circHIPK3 [26], hsa_circ_0000615 [27], and mm9_circ_012559 [28]. CircRNAs have also been suggested to exert their function through-interaction with RNA binding proteins (RBPs), e.g., circ-Foxo3 [29] circ-MBL (muscleblind) [30]; and by modulating mRNA stability by circRNA–mRNA duplex formation, e.g., ciRS-7–CDR1 [31], circ-RasGEF1B-ICAM-1 [32]. Additionally, circRNAs also regulate the transcription of their host genes. For example, circ-EIF3J, circ-PAIP2, ci-ankrd52, and ci-sirt7 are involved in regulating the transcription of their host genes through interaction with RNA Pol II [33,34]. Interestingly, some circRNAs, such as circ-ZNF609 and circMbl3, are translated into proteins [35,36].

A large number of studies reported dysregulation of circRNAs in various cancers, including gastric [37,38], colon [39,40], lung [41,42], leukemia [43], and hepatocellular carcinomas [44,45,46]. circRNAs are also implicated in type 1 and type 2 diabetes and their related complications [47,48,49,50,51]. For example, Shan K et al. [47] studied the role of circRNAs in diabetic retinopathy and observed significant upregulation of circHIPK3 in diabetic retinas and retinal endothelial cells. circHIPK3 silencing in vivo decreased retinal acellular capillaries, vascular leakage, and inflammation and, overall, led to improved retinal vascular dysfunction [47]. The authors further demonstrated that circHIPK3 acted as an endogenous sponge to sequester and inhibit miR-30a-3p activity. In another study, Circ_0054633 was identified as a potential diagnostic biomarker of pre-diabetes and type 2 diabetes in peripheral blood [48]. circRNA Cdr1as regulated insulin transcription and secretion in islet cells via miR-7 and its targets [24]. Overexpression of Cdr1as increased insulin secretion and content in MIN6 and mouse islet cells, leading to an overall improved β-cell function [24]. The expression of circANKRD36 was upregulated in peripheral blood leucocytes of patients with type 2 diabetes and correlated with chronic inflammation in type 2 diabetics [49], thus suggesting its potential as a biomarker for screening chronic inflammation in type 2 diabetic patients. A number of circRNAs were found aberrantly expressed in the placental villi of gestational diabetes patients compared to controls [50]. Recently, Stoll and colleagues used microarray profiling and identified thousands of circRNAs in human pancreatic islets and confirmed dysregulation of ciRS-7 and circHIPK3 in type 2 diabetes mouse models [51].

Pancreatic islets are a heterogeneous agglomeration of at least four distinct hormone-secreting cell types: β, α, δ, γ, and few ε cells [52,53]. These heterogeneous clusters of cells are essential to the diabetes pathophysiology as they function in synergy and elicit concerted, but distinct, responses to maintain glucose homeostasis in the body. The cellular heterogeneity of pancreatic islets poses its own set of challenges, since each of these cell types are known to have distinct epigenetic and transcriptomic signatures [54,55,56,57,58]. Transcriptome profiling of islets may mask cell-specific transcriptomic signatures, which could offer important cues to advance our molecular understanding of events that lead to islet (dys)function [55,56,57,58,59]. Early RNA sequencing (RNA-seq) based transcriptomic profiling studies have specifically focused on cataloging and characterizing the annotated mRNAs, miRNAs, and lncRNAs using human, mouse, or rat islets and insulin producing β-cell lines [60,61,62]. Nonetheless, no study has been undertaken to profile circRNAs’ abundance within each cell-type of human islets. In this study, we sought to decipher the transcriptional landscape of circRNAs, leveraging published RNA-seq datasets of FACS-sorted human α-, β-, and exocrine cells. We identified a cell type-selective repertoire of circRNAs in these three cell types as well as observed alternate circularization events. The circRNAs identified in this study could serve as an important resource for future studies on β-cell function in relation to both type 1 and type 2 diabetes.

## 2. Results

### 2.1. Wide-Spread Expression of circRNAs in α- and β-Cells

To explore the circRNA transcriptome of different cell-types in pancreatic islets, we used the RNA-seq dataset of Bramswig et al. [56] and Ackermann et al. [57] that contained α-, β-cells, and exocrine cells from eight human donors. This is the only non-polyA+ dataset generated on human islets so far available, which allowed us to identify circRNAs. For α- and β-cells, eight replicates per cell-type were included in the analysis, while for the exocrine cells, only two replicates were available. Each sample had a sequencing depth of at least 30 million reads, sequenced single-end with a length of 100 bases (Table S1). On average, α-, β-, and exocrine samples had 57, 64, and 99 million reads, respectively (Table S1).

**Figure 1:**
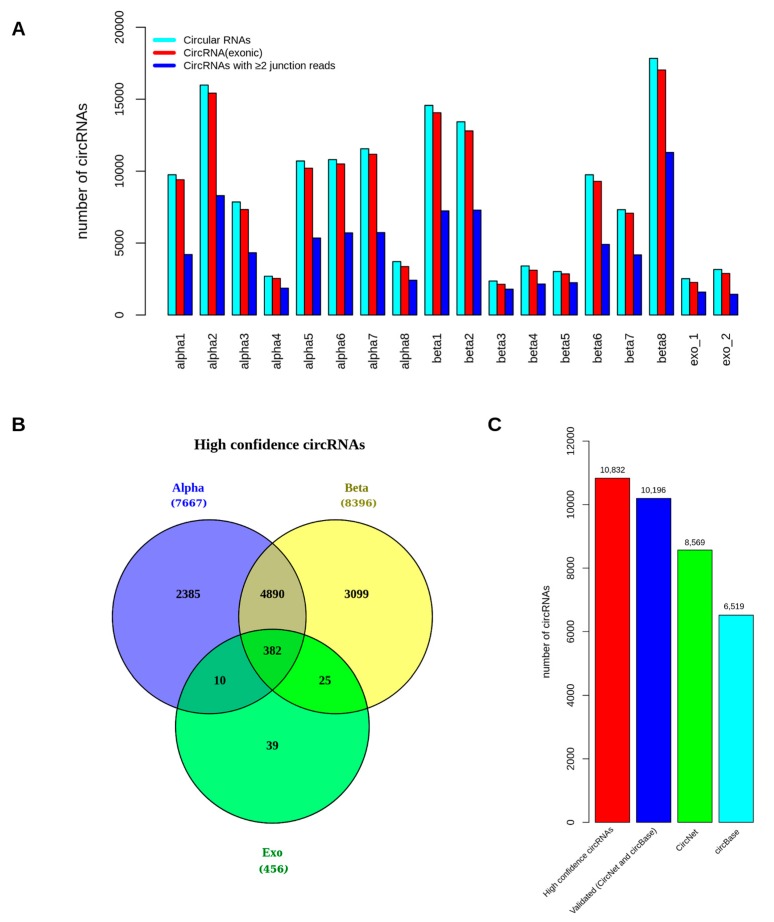
Circular RNA (circRNA) distribution in α-, β-, and exocrine cells. The figure shows (**A**) the total number of circRNAs expressed per sample, (**B**) the overlap between the high confidence circRNAs in α-cell (*n* = 7667), β-cell (*n* = 8396) and exocrine-cell (*n* = 456) and (**C**) the overlap with known circRNAs from CircNet and circBase.

Tophat-fusion and CIRCexplorer2 [63] were used for circRNA identification. CIRCexplorer allows fast and accurate detection of circRNAs by detecting back-spliced junction reads from RNA-seq data and has a very low false positive rate [64,65]. A cutoff of ≥2 junction spanning reads in at least one sample of a specific cell-type population was used to identify circRNAs. The intronic circRNAs candidates were excluded from the analysis and only exonic circRNAs were considered as true candidates. Considering back-splices and selected subset of exonic circRNAs with ≥2 junction spanning reads, the total number of circRNAs expressed per sample is shown (Figure 1A). In total, 17,666, 20,387, and 2575 unique exonic circRNAs (with ≥2 junction reads) were detected in α-, β-cells, and exocrine samples, respectively. Collectively, 28,397 unique circRNAs were detected in all samples from the three cell-types.

To filter out low expressed candidates, we selected for high confidence circRNAs in α-cell (*n* = 7667), β-cell (*n* = 8396), and exocrine-cell (*n* = 456) based on an arbitrary cutoff of expression in ≥2 samples and ≥2 junction spanning reads in each cell-population (Table 1 and Table S2). In total, 10,832 unique high confidence circRNAs (transcribed from 3833 genes) were identified in all samples and 382 candidates were shared among the three cell-types (Figure 1B). These candidates were then compared to already annotated circRNAs as catalogued in circBase [66] and CircNet [67]. A majority (94%) of the high confidence circRNAs identified in our study were found to be annotated by CircNet and circBase with the exact same exonic boundaries (Figure 1C). This overlap further confirms the high true positive rate among the high confidence circRNAs. The shared highly expressed circRNAs in α- and β-cells were identified based on an average of >100 junction spanning reads. Table 2 shows the highly expressed circRNAs, along with their average junction spanning read counts in both α- and β-cells and their corresponding host gene names. The host genes for these highly expressed circRNAs included *MAN1A2*, *RMST*, *HIPK3,* and *RHOBTB3* (Table 2). *MAN1A2*, *HIPK3*, and *RHOBTB3* were also expressed in exocrine cells, although at lower levels (an average of 50–80 junction spanning reads).

### 2.2. Genomic Features of circRNAs in α- and β-Cells

**Figure 2:**
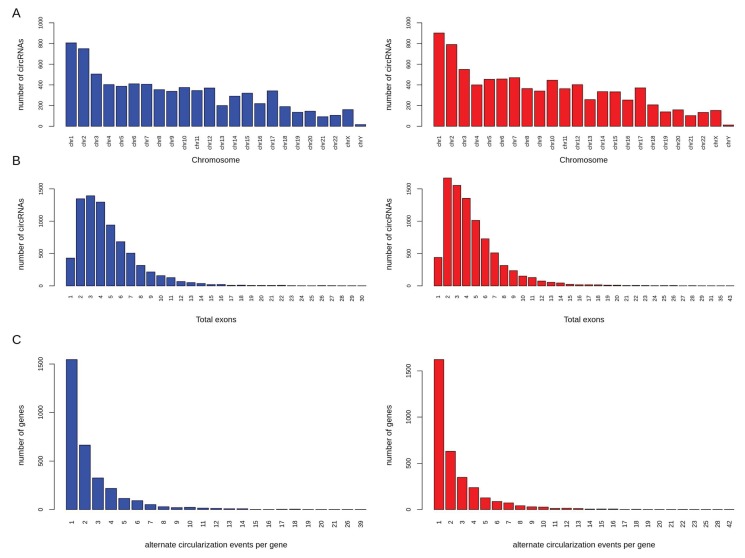
Genomic features of circRNAs in α- and β-cells. The figure shows (**A**) the chromosomal distribution of circRNAs, (**B**) total number of back-spliced exons in circRNAs, and (**C**) number of alternate circularization events per gene in α- (blue) and β- (red) cells.

We next investigated various genomic features of high confidence circRNAs in α- and β-cells. The chromosomal distribution of circRNAs in α- and β-cells (Figure 2A) was found to be similar to other coding and non-coding genes. The α-cell (*n* = 7667) and β-cell (*n* = 8396) high confidence circRNAs were associated to 3141 and 3291 annotated genes, respectively, with two circRNAs per gene on average. The average length for identified circRNAs was 731 and 717 nucleotides for α- and β-cells, respectively. Overall, there was no significant difference in the genomic features of circRNAs in α- and β-cells.

**Figure 3:**
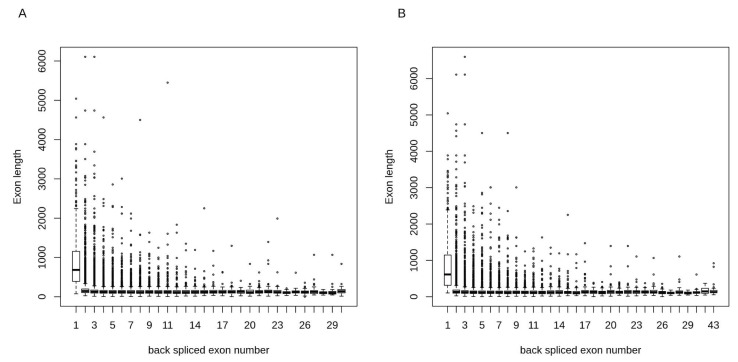
Exon lengths of circRNAs in α- and β-cells. circRNAs with single exons had longer exon lengths in both (**A**) α-cells and (**B**) β-cells as compared to circRNAs with multiple exons.

Around 42% of circRNAs in α- and β-cells contained 1 to 3 exons (Figure 2B). The total number of exons for circRNAs ranged from 1 to 30 and 1 to 43 in α- and β-cells, respectively. We calculated the exon lengths for each circRNA and found that circRNAs with single exons had longer exon length in both α- and β-cells as compared to circRNAs with multiple exons (Figure 3). This suggests that for the back-splicing process, a certain length might be required to maximize exon circularization. Indeed, a similar observation in relation to exon lengths has been reported by Song et al. [68]. 

### 2.3. Alternative Circular Isoforms

Alternative circularization events per gene were detected for both α- and β-cells. In β-cells, 1 to 42 alternate circular isoforms per gene were detected with 68% of the host genes having a maximum of 2 alternate circular isoforms (Figure 2C). In the case of α-cells, a similar trend was observed with 1 to 39 alternate circular isoforms per gene and 70% of the host genes with up to 2 alternate circular isoforms (Figure 2C). In both α- and β-cells, approximately 30% of the genes generated only a single circular variant. The host gene, *PTK2*, had the highest number of alternate circular isoforms with 39, 42, and 2 isoforms in α-cells, β-cells, and exocrine cells, respectively. The host gene, *MAN1A2*, with the highly expressed circRNA candidate, also had other alternate isoforms, (7, 10, and 5 isoforms in α-, β-, and exocrine cells, respectively). These results highlight that alternate circular isoforms are highly abundant in α- and β-cells.

### 2.4. Differentially Expressed circRNAs in β-Cells

**Figure 4:**
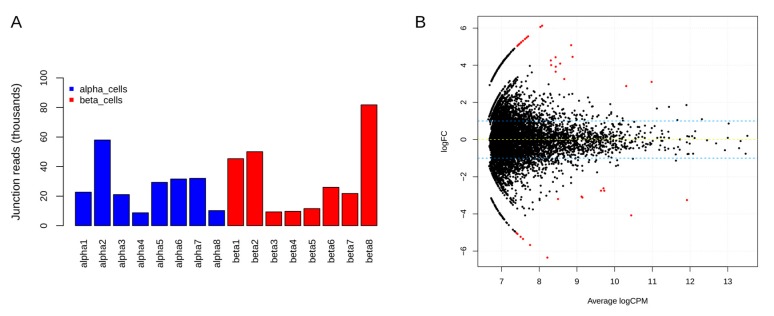
Distribution of junction spanning reads and smear plot of differentially expressed circRNAs. (**A**) Total circRNA junction spanning reads per sample for the high confidence circRNA candidates in α- and β-cells; (**B**) A smear plot highlighting differentially expressed candidates (as red dots) in β-cells when compared to α-cells.

To identify differentially expressed circRNAs in β-cells compared to α-cells, circRNAs were filtered based on expression in at least 25% of the samples and ≥2 junction spanning reads. In total, 6566 circRNA candidates were retained in α- and β-cell libraries after filtering. For most of the samples, on average of 25,000 and 30,000 junction reads were observed in α- and β-cells, respectively (Figure 4A). The differentially expressed circRNAs were identified using a criteria of abs(log2FC) = 1 and adjusted *p*-value < 0.05. Figure 4B shows a smear plot of differentially expressed candidates highlighted in red dots. In total, 36 candidates (from 30 host genes) were differentially expressed, and of these, 22 circRNAs were upregulated and 14 were downregulated in β-cells (Table 3).

circRNA-TGFBR3 (provisional id: 1:91861470:91861644) was the most highly up-regulated circRNA in β-cells with a log2FC of 6.14. Previous studies have shown that *TGFBR3* is highly β-cell selective [57]. Moreover, multiple studies have found circRNA-TGFBR3 expressed in different cell-lines and tissues (CircNet ID: hsa-circ-TGFBR3.25 and circBase ID: hsa_circ_0006622). Out of the two circular isoforms for *TGFBR3* detected in β-cells, 1:91861470:91861644 was the most highly expressed.

**Figure 5:**
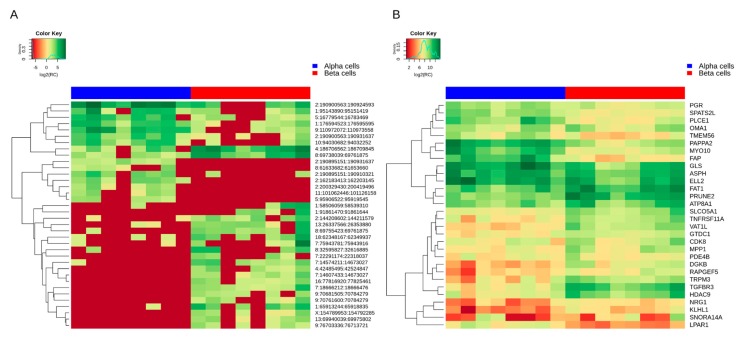
Expression profiles of differentially expressed candidates. Heatmap of normalized expression (log2 RC) of differentially expressed circRNAs (**A**) and their host mRNAs (**B**) in α- and β-cells.

We next investigated how expression levels of differentially expressed circRNAs correlate with expression profiles of their corresponding mRNAs. Figure 5 shows the expression patterns of the 36 differentially expressed circRNAs and their corresponding 30 host mRNAs. The Pearson’s correlations (*r*) were calculated for circRNAs and their host genes using log transformed read counts (logRC). On average, moderate positive correlations (*r* = 0.65) were observed between linear and circRNAs isoforms in combined α- and β-cell samples (Table S3). As shown in the heatmap, both α- and β-cell circRNAs show highly selective expression patterns as compared to their linear forms, which were expressed across the two cell-types. Based on this observation, we focused on cell-specific correlations. For up and down-regulated circRNAs, β-cell and α-cell samples were used, respectively, to compute cell-specific correlations. Moderate positive correlations (*r* = 0.5) were observed for both up and down-regulated circRNAs. Among the up-regulated circRNAs, five out of 11 host gene vs. circRNA correlations were significant (*p*-value < 0.05, *r* ≈ 0.7). In the case of down-regulated circRNAs, six out of 19 host gene vs. circRNAs correlations were significantly positive (*r* ≈ 0.8).

### 2.5. Selectively Expressed circRNAs in α- and β-Cells

Selectively expressed circRNAs, including multiple isoforms from specific gene loci, were identified in α-, β-, and exocrine cells. The circRNA exon-boundaries were intersected in the three cell-types, and those candidates that overlapped by a maximum of 1 bp were excluded. To filter lowly expressed candidates, only those candidates expressed in at least 50% of the samples in each cell-type were considered. Further, circRNAs were filtered by excluding those loci where an alternate circRNA isoform was present in another cell-type. Based on this analysis, 392, 417, and 57 selectively expressed circRNAs were identified in α-, β-, and exocrine cells, respectively. Table 4 shows the number of selectively expressed circRNAs and their corresponding host genes. The top highly and selectively expressed circRNAs and their features are highlighted (Table 5).

Leveraging on the integrated ATAC-seq and RNA-seq analysis by Ackermann et al [57], we classified highly selective circRNAs based on the most highly and selectively expressed transcripts in α- and β-cells. In their analysis, 33 α-cell-selective and 35 β-cell-selective transcripts were identified as the most highly and selectively expressed (defined as ≥10-fold expression difference between α- and β-cells, with false discovery rate (FDR) < 0.05). Several of these α- and β-cell enriched loci were found among our selectively expressed circRNAs (Table 6). These included *TGFBR3,* which is highly β-cell selective, and *FAP*, *SYTL5, PTPRT, STK32B,* and *BVES,* which are highly α-cell selective transcripts. These six loci and their respective circRNAs were labeled highly selective (Table 6).

### 2.6. Functional Annotation of α- and β-Cell Selective circRNAs

Gene ontology (GO) based functional annotation analysis was performed on the host genes that produce α- and β-cell selective circRNAs. In total, 359 host genes for β-cell selective circRNAs and 343 host genes for α-cell selective circRNAs were included in the analysis (referred to as cluster 1 and cluster 2).

**Figure 6:**
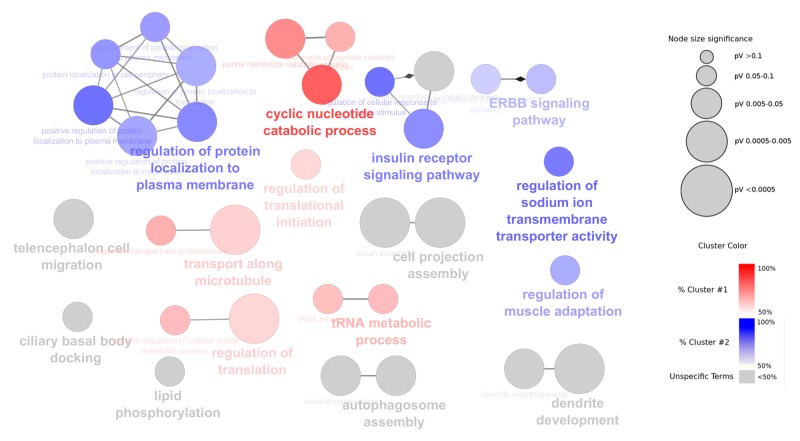
Enriched gene ontology (GO) terms for α- and β-cell selective circRNA host genes. GO terms (biological process) associated with Cluster 1 (β-cell) circRNA host genes are shown in red, Cluster 2 (α-cell) circRNA host genes in blue, and common terms for both clusters are shown in grey.

For both clusters, common as well as cluster-specific GO biological processes (BP) terms were identified. Overall, cluster 1 and 2 showed enrichment for 11 and 12 terms, respectively. We also identified 10 enriched GO-terms common between the two clusters (as shown in grey nodes in Figure 6). Similar GO-terms were grouped and one term for each group based on the *p*-value significance was selected and highlighted in bold (Figure 6). The α-cell specific GO-terms included regulation of protein localization to plasma membrane, insulin receptor signaling, ERBB signaling, and regulation of sodium ion transmembrane transporter activity. In the case of β-cells, five groups of β-cell specific GO-terms were observed that included cyclic nucleotide catabolic processes, regulation of translation, tRNA metabolic processes, regulation of translation initiation, and transport along microtubule. The unspecific (common terms) for both cell-types included cellular response to insulin stimulus pathway, cell projection assembly, lipid phosphorylation, and autophagosome assembly. Of note, since the significant GO terms identified in the analysis reflect the host gene function, the relative contribution of cell-selective expression of circRNAs to the significant GO terms still remains to be established.

**Figure 7:**
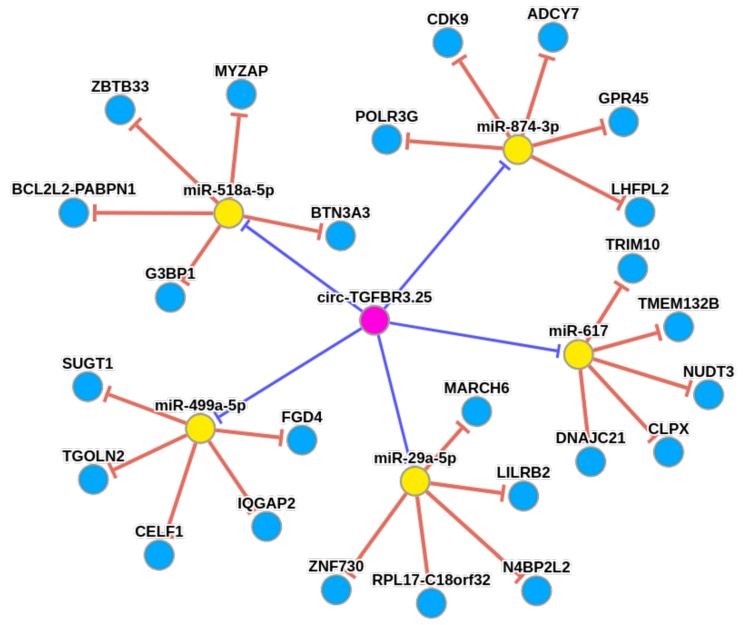
circ-TGFBR3.25 with sponge effect on miRNAs as predicted by CircNet. Yellow nodes represent miRNAs, blue nodes represent genes, and pink nodes represent circRNA. Red edges represent negative regulation of miRNA to the target gene; blue edges represent negative regulation of circRNA to miRNAs.

For the highly β-cell selective circRNA-TGFBR3, we created a circRNA-mRNA-miRNA network using the CircNet database [67]. As shown (Figure 7), circRNA-TGFBR3 might negatively regulate miR-29a-5p, miR-518a-5p, miR-499a-5p, miR-617, and miR-874-3p. Only the top five miRNAs with perfectly aligned miRNA binding sites found on the circRNA sequence are shown (Figure 7). Using the published miRNA expression profiles of human islets and FACS sorted α- and β-cells, the expression of circRNA-TGFBR3 associated miRNAs was evaluated [69,70]. Based on the miRNA-seq profiles from [69], two miRNAs, miR-29a-5p and miR-874-3p, were confirmed to be expressed in both human islets and β-cells (averaged read counts in β-cells: 52 and 1583, respectively). The qPCR profiling of miRNAs in FACS sorted β-cells [70] also confirmed expression of both miR-29a-5p and miR-874-3p in β-cells (average CT value of 28 and 30, respectively). The miRNA binding site for hsa-miR-29a-5p within circ-TGFBR3 is shown as Figure S2.

## 3. Discussion

In both type 1 and type 2 diabetes, islet dysfunction is a key feature. Genome wide association studies have shown that most risk loci in type 2 as well as a substantial part in type 1 diabetes are implicated in islet function [71,72], suggesting that deranged islet cell function is a key culprit in diabetes. Despite many years of intense research, the exact role of the different islet cell populations in islet pathophysiology remains elusive. A deeper understanding of the islet anatomy and how this affects type 1 and type 2 diabetes development is likely to inform the pathogenesis and be important for the development of new treatment modalities.

Recent studies have identified α- and β-cell selective transcriptomic signatures in human islets [54,55,56,57,58]. Various regulatory elements, including islet transcription factors and non-coding RNAs, including lncRNAs, have been reported as essential components for the active maintenance of adult β-cell identity as well as function [73,74,75]. It is plausible that circRNAs might play similar roles in maintaining the β-cell identity and function. Recently, Stoll et al. detected ≈3000 circRNAs in human islets using a microarray-based approach that covered only a fraction of previously annotated circRNAs [51]. In this study, we detected ≈10,000 circRNAs in human α-, β-, and exocrine cells using published high-depth RNA-seq datasets. A majority of the detected circRNAs were mainly expressed in α, and β-cells, as compared to exocrine cells. This observation agrees with other studies reporting enrichment of circRNAs in neuro-endocrine tissues [17,18,19,20]. However, it cannot be ruled out whether the low number of circRNAs detected in exocrine cells in this study is likely due to the small sample size (*n* = 2) compared to endocrine cells (*n* = 8).

The most highly expressed circRNAs identified in this study included *MAN1A2* and *HIPK3*, both exhibiting alternate circular isoforms in the three cell-types under study. Previously, expression of linear *HIPK* 3 has been found to be decreased in type 2 diabetes patients and in ob/ob and db/db mice [51,76]. Moreover, expression of circ-HIPK3 was also decreased in islets of db/db mice and knockdown of circ-HIPK3 resulted in increased apoptosis and decreased β-cell proliferation [51]. It still remains to be elucidated whether this decrease in circ-HIPK3 expression is associated with β-cell survival in human islets or human β-cell-line.

Most of the highly-expressed circRNAs in different tissues and cell-lines have been reported to exist as single isoforms [77]. Only a small fraction of circRNAs are generated in multiple isoforms, with a majority of these isoforms being expressed at low levels [8,77,78]. These findings suggest that the majority of host genes produce only one or two highly expressed circRNA isoforms with functional implications. In both α- and β-cells, 30% of the genes produced single circular isoforms. Host genes associated with the most number of alternate circularization events as well as those producing single isoforms warrant further investigations to inform our understanding of circRNA’s regulatory functions.

The differential expression analysis identified 14 downregulated and 22 upregulated circRNAs in β-cells compared to α-cells. The highly upregulated circRNAs in β-cells included *TGFBR3* and *HDAC9* while the highly downregulated circRNAs included *FAP* and *GLS*. Of note, most of the differentially expressed circRNAs did not show any significant correlation with their linear transcripts. Previous studies have reported similar findings with no significant correlations between linear and circRNAs isoforms in different tissues [14,15]. Alternative methods targeted to specific circRNAs (e.g., custom microarrays or RNAase-R treatment prior to RNA-seq) might provide more accurate estimates for the low abundance, differentially expressed circRNAs.

Although total RNA-seq allows profiling of both coding and non-coding transcripts, including circular forms, low abundant transcripts are often missed due to a low read depth [79]. The numbers of circRNAs identified in our study are far from complete as conceivably low abundant circRNA transcripts were most likely missed. To overcome this limitation, future studies are warranted to profile circRNAs based on enrichment methods, for example, RNase R treatment followed by Polyadenylation and poly(A)+ RNA Depletion (RPAD) [80].

We postulate that the selectively expressed circRNAs identified in this study, including circ-TGFBR3 (provisional id: 1:91861470:91861644), a highly β-cell selective circRNA, might be involved in maintaining the cell identity and regulating cell-specific processes. Importantly, *TGFBR3* locus encodes the transforming growth factor (TGF)- β type III receptor that often functions as a co-receptor with other TGF-β receptor superfamily members and is involved in TGF-β signaling. A recent study showed a novel role of TGF-β signaling in increased β-cell replication [81]. Inhibition of TGF-β signaling induced β-cell replication in human islets and adult mice. We explored the potential miRNA-sponge effect of circRNA-TGFBR3 and found multiple binding sites for several miRNAs. Two of the top five miRNAs in circRNA-TGFBR3-miRNA network miR-29a-5p and miR-874-3p have been found to be expressed in human islets and also in α- and β-cells [69,70]. A discrepancy in the expression of miR-874-3p by miRNA-seq and qPCR based profiling highlights the technical variation of using different methods. The above two studies detected 141 (out of 667 miRNAs screened by qPCR in six human pancreases) and 346 (small RNA-seq, three preparations) miRNAs expressed in β-cells. More than 2000 human miRNAs are annotated so far, therefore, more updated miRNA expression profiling datasets for human α- and β-cells are needed for better understanding of circRNA–miRNA networks. Interestingly, miR-29a has previously been found upregulated in INS-1E β-cells by glucose, leading to decreased glucose-stimulated insulin-secretion [82]. These data suggest that the down regulation of miR-29a by circ-TGFBR3 might directly affect β-cell function. However, the circRNA-TGFBR3-miRNA-mRNA network needs to be experimentally validated.

Taken together, these newly identified cell-selective circRNAs in human islets offer new avenues to explore their functional significance in maintaining β-cell function. Furthermore, the effect of these cell-selective circRNAs on β-cell proliferation, apoptosis, and insulin secretion also warrants further investigations.

## 4. Materials and Methods

### 4.1. circRNA Identification and Analysis

Total RNA-Seq datasets of FACS sorted human α-, β-, and exocrine cells from 8 diseased organ donors were retrieved from GEO (GSE50386 and GSE76268). The dataset included libraries that were single-end sequenced to 100bp on an Illumina hiSeq2000. The SRA accession numbers for all the samples, sample descriptions, and library sizes are listed in Table S1.

The raw fastq files were trimmed, cropped, and adapters removed using Trimmomatic v.0.36 [83]. A two-step mapping strategy was used as previously described [78] to identify circRNAs. The filtered reads were first aligned to a human reference genome (GRCh38) using Tophat2.1 [84] with the following parameters: Library-type = fr-firststrand, no-coverage-search, m 2, p 10. The Tophat-unmapped reads were then extracted and aligned to a human reference genome (GRCh38) with Tophat-Fusion [85] using the following parameters: --fusion-search --keep-fasta-order --bowtie1 --no-coverage-search. The candidate back-spliced junction reads were extracted from Tophat-Fusion alignment and re-aligned against known gene annotations using CIRCexplorer2 (v.2.2) [63]. Intronic circRNA (ciRNA) candidates were discarded from the downstream analysis and only exonic circRNAs with ≥2 junctions spanning reads in at least 1 sample were retained for further analysis. circRNAs expressed in ≥2 samples with ≥2 junctions spanning reads were categorized as high confidence circRNAs in each cell-type population. The junction read counts were normalized to log2 transformed read counts (log2 RC).

For quantification of linear mRNA species, the reads after pre-processing steps (that included trimming and adapter removal) were aligned to a human genome (GRCh38) using tophat 2.1 [84] using the following parameters: Library-type = fr-firststrand, no-coverage-search, m 2, p 10. The total number of reads remaining after QC and percentage of aligned reads for each sample are provided (Table S1). The raw read counts at gene level were calculated using htseqcount [86] and further normalized to counts per million (CPM) and log2 RC in EdgeR [87]. At the transcript level, Cufflinks v2.2.1 [88] was used to calculate FPKM values. A cutoff of FPKM >1 and expression in at least 2 samples in each cell-type was used to filter low-expressed transcripts. The distribution of protein-coding and non-coding transcripts expressed in α-, β-, and exocrine cells is shown in Figure S1.

### 4.2. Differential Expression Analysis

The circRNAs differentially expressed in β-cells compared to α-cells were identified using the EdgeR package in R [79]. For this analysis, circRNAs expressed in at least 25% of the samples and ≥2 junction spanning reads were retained. TMM normalization was applied to this dataset to account for compositional difference between the libraries. Differentially expressed circRNAs were identified by likelihood ratio test (glmLRT) in EdgeR using a cutoff of adjusted *p*-value < 0.05 and absolute log2 fold change (log2 FC) = 1. All the distribution plots, heatmaps, and graphs were generated in R. The Pearson’s correlations (*r*) were calculated for circRNAs and their host genes using log transformed read counts (log2RC). We computed both combined as well as cell-specific correlations for α- and β-cell samples. Cell-specific correlations were computed for the up and down-regulated candidates using β-cell and α-cell samples, respectively.

### 4.3. Functional Annotation of circRNAs

The functional annotation of α- and β-cell selective circRNAs was performed using the ClueGO plugin in Cytoscape [89]. The host genes for α- and β-cell selective circRNAs were used as proxy for functional annotation analysis based on GO-BP categories in ClueGO with default parameters. All human genes were used as background. The *p*-values were adjusted for multiple correction using the BH method. The minimum number and minimum percentage of genes to be included in a group was 5 and 5%, respectively. Similar GO-BP terms were grouped into clusters based on KappaScore grouping with the following parameters: Kappa Score Threshold = 0.5, Sharing Group Percentage = 50%.

### 4.4. miRNA-circRNA Regulatory Network

We created circRNA–miRNA interaction networks using CircNet [67]. CircNet performs micRNA sponge detection analysis by iteratively searching the circRNA isoforms sequences for miRNA target sequences with perfect complementarity. The occurrence of miRNA target seeds in circRNA isoforms are examined and normalized by isoform length. The significance of interactions is evaluated by referring to the background distribution of miRNA seeds in all transcripts, and only circRNA-miRNA interactions with *p* < 0.001 are considered significant. CircNet combines the target genes of miRNAs from miRTarBase [90], which are also included in networks.

## Figures and Tables

**Table 1:** High confidence circular RNAs (circRNAs) in α-, β-, and exocrine cells. The high confidence circRNAs were selected based a criterion of expression in ≥2 samples and ≥2 junction spanning reads.

	circRNAs	Genes
α-Cells	7667	3141
β-Cells	8396	3291
Exocrine-cells	456	388
Common circRNAs	382	332
Total	10,830	3833

**Table 2:** Highly expressed circRNAs in α- and β-cells. The highly expressed candidates with an average of >100 junction spanning reads in α- and β-cells are listed along with their annotations and expression levels.

circRNA ID	Gene Name	Average Junction Reads (α-Cells)	Average Junction Reads (β-Cells)	circBase ID
1:117402185:117420649	*MAN1A2*	267	354	hsa_circ_0000118
12:97492460:97561047	*RMST*	354	220	-
11:33286412:33287511	*HIPK3*	263	286	hsa_circ_0000284
4:152411302:152412529	*FBXW7*	285	241	hsa_circ_0001451
5:95755395:95763620	*RHOBTB3*	137	307	hsa_circ_0007444
4:143543508:143543972	*SMARCA5*	180	228	hsa_circ_0001445
7:100023418:100024307	*ZKSCAN1*	220	177	hsa_circ_0001727
10:31908171:31910563	*ARHGAP12*	174	167	hsa_circ_0000231
7:24623665:24668660	*MPP6*	134	144	hsa_circ_0001686
18:9182381:9221999	*ANKRD12*	101	123	hsa_circ_0000826
2:50921868:50925955	*NRXN1*	106	110	hsa_circ_0001004

**Table 3:** Differentially expressed circRNAs in β-cells compared to α-cells. In total, 14 downregulated and 22 upregulated circRNAs were identified in β-cells using a cutoff of logFC ≥ 1 and adjusted *p*-value < 0.05.

circRNA	Gene Name	Isoform Name	logFC	*p*-Value	adj. *p*-Value
**Down-Regulated**					
2:162183413:162203145	*FAP*	ENST00000627638	−6.34	4.40 × 10^−8^	7.23 × 10^−5^
2:190895151:190931637	*GLS*	ENST00000320717	−5.67	5.97 × 10^−6^	3.92 × 10^−3^
2:200329430:200419496	*SPATS2L*	ENST00000409718	−5.34	4.09 × 10^−5^	1.22 × 10^−2^
11:101062446:101126158	*PGR*	ENST00000325455	−5.22	7.83 × 10^−5^	1.98 × 10^−2^
5:95906522:95919545	*ELL2*	ENST00000237853	−5.07	1.77 × 10^−4^	3.53 × 10^−2^
8:61633682:61653660	*ASPH*	ENST00000379454	−5.03	2.26 × 10−4	4.24 × 10^−2^
9:110972072:110973558	*LPAR1*	ENST00000374431	−4.07	2.45 × 10^−9^	1.61 × 10^−5^
2:190900563:190924593	*GLS*	ENST00000320717	−3.25	4.03 × 10^−8^	7.23 × 10^−5^
2:190895151:190910321	*GLS*	ENST00000320717	−3.19	1.65 × 10^−4^	3.39 × 10^−2^
10:94030682:94032252	*PLCE1*	ENST00000260766	−3.11	2.67 × 10^−5^	1.03 × 10^−2^
2:190900563:190931637	*GLS*	ENST00000320717	−3.06	3.98 × 10^−5^	1.22 × 10^−2^
5:16779544:16783469	*MYO10*	ENST00000274203	−2.74	5.29 × 10^−5^	1.51 × 10^−2^
1:176594523:176595595	*PAPPA2*	ENST00000367661	−2.74	5.93 × 10^−5^	1.56 × 10^−2^
1:95143890:95151419	*TMEM56*	ENST00000370203	−2.61	9.18 × 10^−5^	2.08 × 10^−2^
**Up-regulated**					
1:91861470:91861644	*TGFBR3*	ENST00000212355	6.14	3.16 × 10^−7^	3.46 × 10^−4^
7:18666212:18666476	*HDAC9*	ENST00000461159	6.07	4.91 × 10^−7^	4.24 × 10^−4^
7:14607433:14673027	*DGKB*	ENST00000399322	5.56	1.41 × 10^−5^	7.13 × 10^−3^
16:77816920:77825461	*VAT1L*	ENST00000302536	5.49	2.17 × 10^−5^	8.90 × 10^−3^
4:42485495:42524847	*ATP8A1*	ENST00000381668	5.42	3.33 × 10^−5^	1.18 × 10^−2^
9:70761600:70784279	*TRPM3*	ENST00000377110	5.33	5.62 × 10^−5^	1.54 × 10^−2^
9:70681505:70784279	*TRPM3*	ENST00000377110	5.25	9.04 × 10^−5^	2.08 × 10^−2^
13:69940039:69975802	*KLHL1*	ENST00000377844	5.23	9.71 × 10^−5^	2.12 × 10^−2^
7:22291174:22318037	*RAPGEF5*	ENST00000344041	5.15	1.43 × 10^−4^	3.02 × 10^−2^
9:76703336:76713721	*PRUNE2*	ENST00000376718	5.11	1.85 × 10^−4^	3.58 × 10^−2^
1:65913244:65918835	*PDE4B*	ENST00000329654	5.09	3.28 × 10^−8^	7.23 × 10^−5^
X:154789953:154792285	*MPP1*	ENST00000369534	5.05	2.37 × 10^−4^	4.33 × 10^−2^
7:75943781:75943916	*SNORA14A*	ENST00000364773	4.46	3.13 × 10^−7^	3.46 × 10^−4^
8:69755423:69761875	*SLCO5A1*	ENST00000260126	4.44	3.25 × 10^−6^	2.37 × 10^−3^
7:14574211:14673027	*DGKB*	ENST00000399322	4.27	1.62 × 10^−5^	7.60 × 10^−3^
13:26337566:26353880	*CDK8*	ENST00000536792	4.10	6.81 × 10^−6^	4.06 × 10^−3^
2:144208602:144211579	*GTDC1*	ENST00000392869	4.02	3.42 × 10^−5^	1.18 × 10^−2^
1:58506059:58539310	*OMA1*	ENST00000371226	3.93	2.17 × 10^−5^	8.90 × 10^−3^
8:32595827:32616885	*NRG1*	ENST00000523534	3.66	4.06 × 10^−5^	1.22 × 10^−2^
18:62348167:62349937	*TNFRSF11A*	ENST00000269485	3.27	8.98 × 10^−5^	2.08 × 10^−2^
4:186706562:186709845	*FAT1*	ENST00000441802	3.11	5.17 × 10^−7^	4.24 × 10^−4^
8:69738039:69761875	*SLCO5A1*	ENST00000260126	2.89	7.85 × 10^−6^	4.29 × 10^−3^

**Table 4:** Selectively expressed circRNAs in human islets. The number of selectively expressed circRNAs and highly selective circRNAs are listed along with the total number of host genes. Highly selective circRNAs are those that originate from the most highly and selectively expressed transcripts in each cell type.

	Selectively Expressed circRNAs		Highly Selective circRNAs	
	circRNAs	Genes	circRNAs	Genes
α-Cell	392	343	7	5
β-Cell	417	359	1	1
Exocrine cells	57	52	-	-

**Table 5:** Top highly and selectively expressed circRNAs for α-, β-, and exocrine cells. The averaged log-transformed junction read counts (log2(RC) are shown as a measure of normalized expression for each circRNA. Other columns include the total number of exons for each circRNA as represented by exon Count, circRNA length, and host gene name and biotype.

Gene Name	circ ID	Exon Count	circRNA Length	log2(RC)	Gene Biotype
**α-cell selective circRNAs**					
*PLCE1*	10:94030682:94032252	1	1570	4.43	protein_coding
*FAP*	2:162183413:162203145	9	822	3.38	protein_coding
*KANK1*	9:710803:713464	1	2661	3.27	protein_coding
*MCU*	10:72715110:72715902	2	165	3.02	protein_coding
*C1orf168*	1:56787174:56792803	3	944	2.95	protein_coding
*FRMPD3*	X:107526581:107533550	3	304	2.93	protein_coding
*PLCE1*	10:94245945:94246621	1	676	2.88	protein_coding
*KIAA1217*	10:24219625:24219909	1	284	2.70	protein_coding
*SYTL5*	X:38033533:38034008	1	475	2.70	protein_coding
*FAM126A*	7:22960255:22991139	9	1019	2.64	protein_coding
*SPG21*	15:64974601:64983593	4	476	2.55	protein_coding
*POU6F2*	7:39204234:39207620	2	321	2.55	protein_coding
*PITPNB*	22:27894554:27914347	6	436	2.52	protein_coding
*PRKCE*	2:46001403:46010517	4	614	2.49	protein_coding
*SPATS2L*	2:200329430:200419496	5	517	2.49	protein_coding
**β-cell selective circRNAs**					
*SNORA14A*	7:75943781:75943916	1	135	4.96	snoRNA
*PDE4B*	1:65913244:65918835	2	351	4.37	protein_coding
*TNFRSF11A*	18:62348167:62349937	2	208	4.21	protein_coding
*NRG1*	8:32595827:32616885	4	402	3.95	protein_coding
*SNORD9*	14:21392149:21392253	1	104	3.63	snoRNA
*DGKB*	7:14574211:14673027	8	738	3.58	protein_coding
*GTDC1*	2:144208602:144211579	2	267	3.48	protein_coding
*SYNE2*	14:63998913:64022863	12	2284	3.39	protein_coding
*HDAC9*	7:18666212:18666476	1	264	3.36	protein_coding
*TNRC6B*	22:40117054:40156182	3	233	3.36	protein_coding
*TGFBR3*	1:91861470:91861644	1	174	3.29	protein_coding
*NRD1*	1:51827795:51834170	2	228	3.29	protein_coding
*GLCCI1*	7:8003907:8060248	4	509	3.21	protein_coding
*ROR1*	1:64009304:64050716	3	391	3.04	protein_coding
*TSNAX*	1:231537212:231542611	2	246	2.88	protein_coding
**Exocrine-cell selective circRNAs**					
*SNORD26*	11:62855291:62855366	1	75	5.82	snoRNA
*PNLIPRP2*	10:116625945:116627874	3	373	4.78	polymorphic_pseudogene
*CFTR*	7:117504252:117559655	10	1531	4.29	protein_coding
*C15orf38-AP3S2*	15:89903215:89910819	4	580	4.29	protein_coding
*PNLIPRP2*	10:116625945:116631357	5	613	4.21	polymorphic_pseudogene
*RAB3D*	19:11335446:11335783	2	244	4.13	protein_coding
*SEL1L*	14:81492479:81495137	2	126	3.81	protein_coding
*TRHDE*	12:72562164:72575542	5	533	3.64	protein_coding
*TRHDE*	12:72562852:72575542	4	467	3.52	protein_coding
*SLC7A6*	16:68266592:68266721	1	129	3.09	protein_coding
*IKBKB*	8:42325969:42329214	2	219	3.09	protein_coding
*SLC43A1*	11:57491223:57491862	3	322	3.09	protein_coding
*C3orf52*	3:112109542:112113145	2	253	2.91	protein_coding
*ARF4*	3:57583897:57584464	2	191	2.91	protein_coding
*SNORD58B*	18:49491663:49491729	1	66	2.91	snoRNA

**Table 6:** Highly selective α- and β-cell circRNAs are highlighted based on the overlap with integrated ATAC-seq and mRNA-seq analysis from [57].

α-Cell-Selective circRNAs				
Gene Name	CHR	circ ID	circBase or CircNet Id	log2(RC)
*FAP*	2	2:162183413:162203145	hsa-circ-FAP.15	3.4
*SYTL5*	X	X:38033533:38034008	hsa-circ-SYTL5.3	2.7
*PTPRT*	20	20:42448219:42472562	hsa-circ-PTPRT.2, hsa_circ_0060424	1.9
*PTPRT*	20	20:42350627:42352285	hsa-circ-PTPRT.5	1
*PTPRT*	20	20:42756461:42791466	hsa-circ-PTPRT.15	0.2
*STK32B*	4	4:5139904:5168450	hsa-circ-STK32B.6, hsa_circ_0004536	0.5
*BVES*	6	6:105115685:105125578	hsa-circ-BVES.2, hsa_circ_0077527	0.3
**β-cell-selective circRNAs**				
*TGFBR3*	1	1:91861470:91861644	hsa-circ-TGFBR3.25, hsa_circ_0006622	3.3

## Supplementary Materials

The following are available online at http://www.mdpi.com/2311-553X/4/4/38/s1, Table S1: Datasets retrieved and analyzed for circRNAs in FACS sorted human islets, Table S2: Full list of high confidence circRNAs detected in α-, β- and exocrine cells. Table S3: Pearson’s correlation between differentially expressed circRNAs and host mRNA. Figure S1: Distribution of protein-coding and non-coding transcripts expressed in α-, β- and exocrine cells. Figure S2: miRNA binding sites for hsa-miR-29a-5p within circRNA-TGFBR3 (ID: 1:91861470:91861644).
