# Supplementary material for: Cell Type-Selective Expression of Circular RNAs in Human Pancreatic Islets
Source: Noncoding RNA. 2018 Nov 27;4(4):38. doi: 10.3390/ncrna4040038 (PMC6316812; doi:10.3390/ncrna4040038)
Supplement: Supplementary file 1 [file ncrna-04-00038-s001.zip › Supplementary Figures.docx]

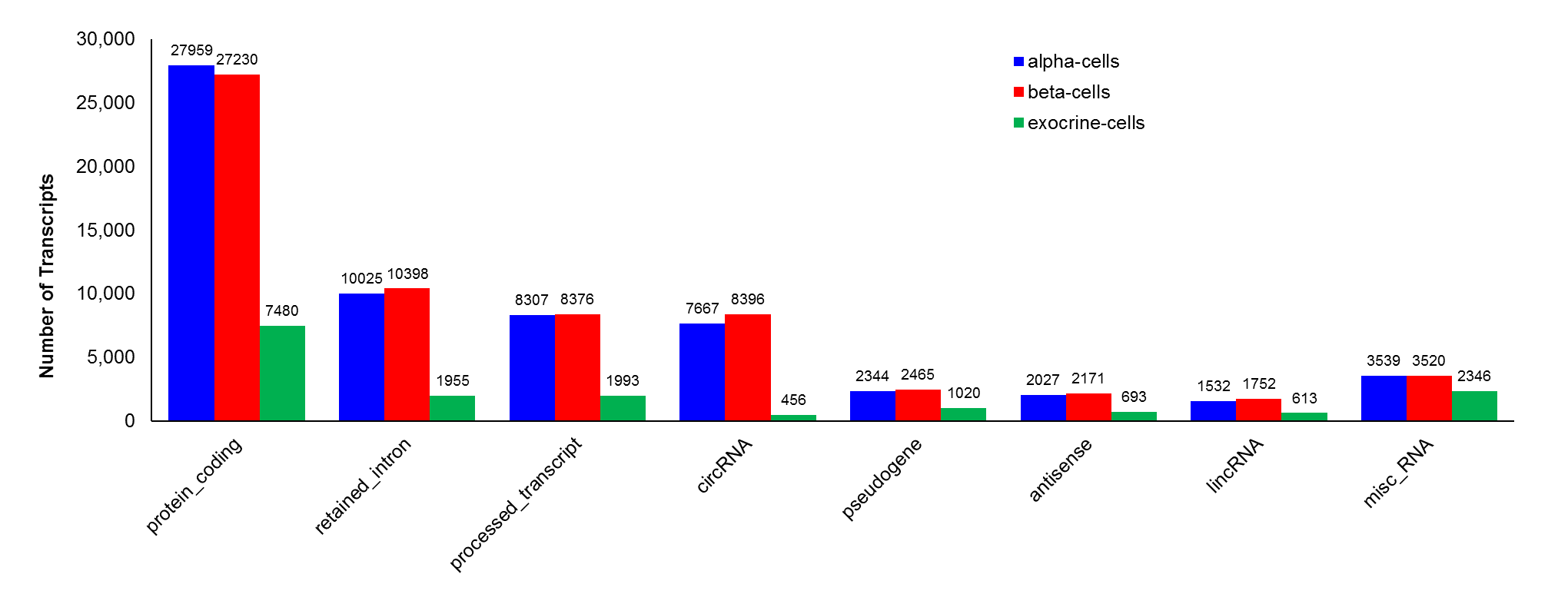


**Figure S1: Distribution of protein-coding and non-coding transcripts expressed in α, β and exocrine cells.** The figure shows distribution of protein-coding and non-coding transcripts expressed in α, β and exocrine cells. The transcript biotypes are based on Gencode v24 annotations. The total number of high confidence circRNAs identified in this study are also included. The misc_RNA category includes snoRNA, snRNA, miRNA, sense overlapping, sense intronic, TEC (To be confirmed) and other unannotated ncRNA transcripts.

**
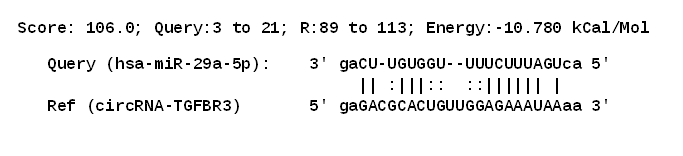
**

**Figure S2: miRNA binding sites for hsa-miR-29a-5p within circRNA-TGFBR3 (ID: 1:91861470:91861644).** The miRNA binding site prediction analysis was performed using miRanda [1] with default parameters.

1. A.J. Enright, B. John, U. Gaul, T. Tuschl, C. Sander, D.S. Marks; (2003) MicroRNA targets in Drosophila; Genome Biology 5(1):R1.
