# Supplementary material for: Cell Type-Selective Expression of Circular RNAs in Human Pancreatic Islets
Source: Noncoding RNA. 2018 Nov 27;4(4):38. doi: 10.3390/ncrna4040038 (PMC6316812; doi:10.3390/ncrna4040038)
Supplement: Supplementary file 1 [file ncrna-04-00038-s001.zip › Table S1.docx]

**Table S1: Datasets retrieved and analyzed for circRNAs in FACS sorted human islets.** The table shows the GEO and SRA IDs of the dataset analyzed for circRNAs. The sample ID from 10 donors is displayed along with their sex and age. The total number of reads remaining after trimming for adapters and initial QC and percentage of surviving reads for each sample are displayed.

| **GEO ID** | **SRA ID** | **Sample Name** | **Sample ID** | **Age** | **Sex** | **Total reads** | **Reads after QC** | **% Survived Reads** |
| --- | --- | --- | --- | --- | --- | --- | --- | --- |
| GSE76268 | SRR3048055 | alpha1 | ABC3413 | 60 | M | 35,792,489 | 34,431,836 | 96.20% |
| GSE76268 | SRR3048056  SRR3048057  SRR3048058 | alpha2 | ABEL098 | 47 | F | 43,868,393 | 42,130,492 | 96.04% |
| GSE76268 | SRR3048059 | alpha3 | CITH068 | 52 | M | 97,704,374 | 90,445,647 | 92.57% |
| GSE76268 | SRR3048060 | alpha4 | CITH070 | 28 | M | 72,405,803 | 66,710,361 | 92.13% |
| GSE76268 | SRR3048061  SRR3048062  SRR3048063 | alpha5 | ICRH025 | 47 | M | 42,776,353 | 40,832,974 | 95.46% |
| GSE76268 | SRR3048064  SRR3048065  SRR3048066 | alpha6 | ICRH039 | 50 | M | 42,026,656 | 40,282,626 | 95.85% |
| GSE76268 | SRR3048067  SRR3048068 | alpha7 | ICRH041 | 28 | M | 44,879,497 | 43,198,256 | 96.25% |
| GSE50386 | SRR958701 | alpha8 | CITH053 | 49 | F | 81,365,373 | 74,526,099 | 91.59% |
|  | SRR3048070 | beta1 | ABEF248 | 60 | F | 52,194,897 | 50,014,527 | 95.82% |
| GSE76268 | SRR3048071  SRR3048072  SRR3048073 | beta2 | ABEL098 | 47 | F | 45,639,061 | 43,771,861 | 95.91% |
| GSE76268 | SRR3048074 | beta3 | CITH053 | 49 | F | 74,954,527 | 67,970,188 | 90.68% |
| GSE76268 | SRR3048075 | beta4 | CITH068 | 52 | M | 78,508,860 | 73,419,573 | 93.52% |
| GSE76268 | SRR3048076 | beta5 | CITH070 | 28 | M | 73,291,067 | 66,922,986 | 91.31% |
| GSE76268 | SRR3048077  SRR3048078  SRR3048079 | beta6 | ICRH025 | 47 | M | 43,471,625 | 41,474,965 | 95.41% |
| GSE76268 | SRR3048080  SRR3048081  SRR3048082 | beta7 | ICRH041 | 28 | M | 36,289,494 | 34,828,877 | 95.98% |
| GSE76268 | SRR3048083 | beta8 | ICRH057 | 40 | M | 114,783,044 | 110,517,071 | 96.28% |
| GSE50386 | SRR958708 | exo1 | CITH053 | 49 | F | 93,127,462 | 85,053,306 | 91.33% |
| GSE50386 | SRR958707 | exo2 | CITH068 | 52 | M | 104,980,114 | 92,630,950 | 88.24% |
