# Supplementary material for: Cell Type-Selective Expression of Circular RNAs in Human Pancreatic Islets
Source: Noncoding RNA. 2018 Nov 27;4(4):38. doi: 10.3390/ncrna4040038 (PMC6316812; doi:10.3390/ncrna4040038)
Supplement: Supplementary file 1 [file ncrna-04-00038-s001.zip › Table S3.docx]

**Table S3: Pearson’s correlation between differentially expressed circRNAs and host mRNA.** The correlations were computed across as well as separately for α and β-cell samples. The significant correlations are highlighted in red. The normalized read counts (log2RC) are shown for linear mRNA in both α and β-cells.

| **Down-regulated CircRNAs** | | | | | | | | |
| --- | --- | --- | --- | --- | --- | --- | --- | --- |
| **CircRNA** | **Genes** | **Pearson's correlation using both cell-types** | | **P-value** | **Pearson's correlation in α-cells** | **P-value** | **log2RC host gene (α-cells)** | **log2RC host gene (β-cells)** |
| 2:162183413:162203145 | FAP | 0.80 | | 1.06E-04 | 0.27 | 0.523 | 10.74 | 6.89 |
| 2:190895151:190931637 | GLS | 0.67 | | 0.004 | 0.59 | 0.122 | 12.26 | 10.4 |
| 2:200329430:200419496 | SPATS2L | 0.94 | | 0.000 | 0.9 | 0.002 | 9.41 | 7.5 |
| 11:101062446:101126158 | PGR | 0.75 | | 0.001 | 0.68 | 0.066 | 9.06 | 8.02 |
| 5:95906522:95919545 | ELL2 | 0.64 | | 0.007 | 0.84 | 0.01 | 12.17 | 11.06 |
| 8:61633682:61653660 | ASPH | 0.29 | | 0.280 | 0.13 | 0.753 | 11.65 | 10.99 |
| 9:110972072:110973558 | LPAR1 | 0.75 | | 0.001 | 0.83 | 0.011 | 7.64 | 4.62 |
| 2:190900563:190924593 | GLS | 0.91 | | 9.85E-07 | 0.81 | 0.014 | 12.26 | 10.4 |
| 2:190895151:190910321 | GLS | 0.72 | | 0.002 | 0.76 | 0.028 | 12.26 | 10.4 |
| 10:94030682:94032252 | PLCE1 | 0.75 | | 0.001 | 0.55 | 0.158 | 9.96 | 7.57 |
| 2:190900563:190931637 | GLS | 0.71 | | 0.002 | 0.8 | 0.018 | 12.26 | 10.4 |
| 5:16779544:16783469 | MYO10 | 0.50 | | 0.049 | 0.37 | 0.36 | 10.92 | 7.94 |
| 1:176594523:176595595 | PAPPA2 | 0.83 | | 7.64E-05 | 0.75 | 0.032 | 11.7 | 8.74 |
| 1:95143890:95151419 | TMEM56 | 0.71 | | 0.002 | 0.65 | 0.079 | 8.4 | 6.59 |
| **Up-regulated circRNAs** | | | | | | | | |
| **CircRNA** | **Genes** | **Pearson's correlation using both cell-types** | **P-value** | | **Pearson's correlation in β-cells** | **P-value** | **log2RC host gene (α-cells)** | **log2RC host gene (β-cells)** |
| 1:91861470:91861644 | TGFBR3 | 0.41 | 0.111 | | -0.32 | 0.446 | 7.44 | 10.79 |
| 7:18666212:18666476 | HDAC9 | 0.84 | 3.83E-05 | | 0.84 | 0.01 | 7.63 | 9.98 |
| 7:14607433:14673027 | DGKB | 0.86 | 1.56E-05 | | 0.82 | 0.013 | 5.45 | 8.08 |
| 16:77816920:77825461 | VAT1L | 0.89 | 3.36E-06 | | 0.64 | 0.088 | 6.45 | 9.07 |
| 4:42485495:42524847 | ATP8A1 | 0.72 | 0.002 | | 0.8 | 0.017 | 9.55 | 10.48 |
| 9:70761600:70784279 | TRPM3 | 0.66 | 0.005 | | 0.54 | 0.165 | 6.44 | 8.97 |
| 9:70681505:70784279 | TRPM3 | 0.63 | 0.009 | | 0.35 | 0.397 | 6.44 | 8.97 |
| 13:69940039:69975802 | KLHL1 | 0.63 | 0.009 | | 0.3 | 0.463 | 4.06 | 6.53 |
| 7:22291174:22318037 | RAPGEF5 | 0.49 | 0.055 | | 0.41 | 0.319 | 5.71 | 7.66 |
| 9:76703336:76713721 | PRUNE2 | 0.72 | 0.002 | | 0.25 | 0.558 | 9.6 | 11.85 |
| 1:65913244:65918835 | PDE4B | 0.54 | 0.029 | | 0.64 | 0.085 | 7.19 | 7.53 |
| X:154789953:154792285 | MPP1 | 0.81 | 1.61E-04 | | 0.72 | 0.043 | 7.17 | 8.41 |
| 7:75943781:75943916 | SNORA14A | 0.02 | 0.932 | | 0.11 | 0.793 | 4.3 | 5.61 |
| 8:69755423:69761875 | SLCO5A1 | 0.70 | 0.002 | | 0.2 | 0.642 | 7.58 | 9.24 |
| 7:14574211:14673027 | DGKB | 0.79 | 2.92E-04 | | 0.88 | 0.004 | 5.45 | 8.08 |
| 13:26337566:26353880 | CDK8 | 0.46 | 0.073 | | 0.04 | 0.925 | 7.56 | 8.45 |
| 2:144208602:144211579 | GTDC1 | 0.62 | 0.010 | | 0.43 | 0.285 | 7.1 | 8.27 |
| 1:58506059:58539310 | OMA1 | 0.07 | 0.790 | | 0.33 | 0.421 | 8.67 | 8.65 |
| 8:32595827:32616885 | NRG1 | 0.23 | 0.401 | | 0.48 | 0.231 | 5.02 | 6.81 |
| 18:62348167:62349937 | TNFRSF11A | 0.67 | 0.005 | | 0.67 | 0.067 | 7.01 | 8.97 |
| 4:186706562:186709845 | FAT1 | 0.76 | 0.001 | | 0.89 | 0.003 | 10.29 | 11.86 |
| 8:69738039:69761875 | SLCO5A1 | 0.72 | 0.002 | | 0.91 | 0.002 | 7.58 | 9.24 |
